# Which Factors Predict L2 Receptive Vocabulary and Expressive Syntax in Bilingual Children from Low-SES Families?

**DOI:** 10.3390/children11101165

**Published:** 2024-09-25

**Authors:** Arianna Bello, Paola Ferraresi, Susanna Pallini, Paola Perucchini, Antonia Lonigro

**Affiliations:** Department of Education, Roma Tre University, 00185 Rome, Italy; pa.ferraresi@gmail.com (P.F.); susanna.pallini@uniroma3.it (S.P.); paola.perucchini@uniroma3.it (P.P.); antonia.lonigro@uniroma3.it (A.L.)

**Keywords:** bilingualism, receptive vocabulary, expressive syntax, phonological processing, language exposure, low SES

## Abstract

Introduction: The objective of the current study was two-fold. First, it aimed to estimate receptive vocabulary and expressive syntax skills in L2 Italian among early sequential/simultaneous bilingual children of migrant single-mother families with very low socioeconomic status (SES). This objective was achieved by matching the participants’ performance with normative data. Secondly, this study aimed to identify which individual and language exposure factors contributed to learning L2 vocabulary and syntax. Methods: Twenty-four early sequential/simultaneous bilingual children (age range = 5.10–12.4 years) and their mothers were enrolled. Mothers answered questions about linguistic biography and demographic information. Children completed Lexical Comprehension, Sentence Repetition, and Non-Word Repetition tasks from the Language Assessment Battery for 4–12-year-olds to, respectively, assess receptive vocabulary, expressive syntax, and phonological processing. Moreover, non-verbal intellectual functioning was evaluated by the Raven’s Test. Results/Discussion: Compared to normative data, 20 children showed lower receptive vocabulary abilities (<−1.5 SD), 24 lower expressive syntax skills (−2DS), and 7 children lower phonological processing (<−1.5 DS). Moreover, L2 phonological processing and the length of L2 exposure in an educational context positively predicted L2 receptive vocabulary as well as L2 expressive syntax skills. To date, performance in L2 among early sequential/simultaneous bilingual children from migrant households and very low SES remains underexplored. Future efforts need to be directed towards the understanding of factors that impact oral competence in L2, considering that these children will also be exposed to written L2 in the school context.

## 1. Introduction

In Italy, the number of bilingual children with a migration background is constantly increasing, and in the 2022/2023 school year they comprised 11% of the children attending school [1]. The general aim of this study is to investigate second language (L2 Italian) acquisition by bilingual children who are exposed to a minority first language (L1) at home through their mothers and to L2 in a pre-school/school or social context, specifically, children growing up in an Italian community and educated exclusively in Italian. As ‘early sequential bilinguals’, they use mainly another language at home and never attended school in another country [2]. In addition, these children come from low-educational-status and no-employment single-parent migrant families with absent fathers. Thus, these families received support from social services.

As previously documented in L2 American English children, both belonging to families with low socioeconomic status (SES) and being exposed to an L1 minority language at home negatively affected L2 acquisition and academic success [3]. More recently, ref. [4] confirmed that bilingual exposure and SES are two environmental factors closely linked to children’s developmental trajectories in several domains from pre-school age.

The main aim of the present study was to explore L2 acquisition in typically developing bilinguals with normal non-verbal cognitive functioning. Based on previous research findings on the language development of bilinguals with and without Developmental Language Disorder [2,5,6,7,8], we used three different types of tasks, target word recognition, sentence repetition, and non-word repetition, which evaluate receptive vocabulary, expressive syntax, and phonological processing, respectively. Although the Language Impairment Testing in Multilingual Settings [9] is recognized as an appropriate tool in assessing bilinguals to identify children with impairments in language development, we did not use it because our sample children were older. Thus, we adopted an instrument that is commonly used in Italy to focus on the strengths in bilinguals and discriminate typical and atypical language acquisition.

As reported in the literature, several individual factors (e.g., age at testing, non-verbal cognitive functioning, phonological processing) and language exposure factors (e.g., length of L2 exposure—age of onset of exposure; L1/L2 current exposure) may predict the L2 receptive vocabulary and syntactic competences in bilingual children [10,11]. Thus, we were interested in understanding how these factors contribute to L2 acquisition specifically in children from low-SES families and L1 minority homes. 

In the following paragraphs, we report a brief review of the recent results on the predictive role of these factors on L2 receptive vocabulary and expressive syntactic skills.

### 1.1. Receptive Vocabulary and Expressive Syntactic Abilities in L2

Traditionally, the L2 receptive vocabulary of bilingual children has been studied by using standardized tests [12], such as the target word recognition task (e.g., the most common is the Peabody Picture Vocabulary Test; PPVT, [13]. Pre-schoolers and school-aged bilingual children show lower L2 receptive vocabulary skills compared to monolingual peers, but their performance reaches those of monolinguals after about three years of L2 exposure [14,15]. Concerning the rate of increase in L2, bilinguals show a faster increase in L2 receptive vocabulary than monolingual peers [16].

Some studies demonstrated that L2 receptive vocabulary scores of bilingual children coming from low-SES families are below two standard deviations with respect to monolingual norms [15]. By examining L1 and L2 vocabulary separately and controlling for SES, bilingual children who learn L2 after L1 (i.e., late sequential bilinguals) score lower on receptive vocabulary tasks compared to monolinguals and simultaneous bilinguals (i.e., children exposed to two languages from birth) (among others [17,18]).

In the bilingual population, L2 expressive syntactic competences have been explored through the use of the sentence repetition task, also known as ‘sentence recall’ or ‘sentence imitation’, which is a very easy task to administer and is usable in different languages [19,20,21]. Studies on sentence repetition accuracy in bilingual and monolingual children did not demonstrate coherent and exhaustive results, perhaps due to factors related to child characteristics, language exposure, and L1/L2 type. Ref. [22] documented lower L2 English sentence repetition accuracy and wider interindividual variability in bilingual children aged from 5 to 8 years with L1 heterogeneous background and variety of L2 English use at home than English-speaking monolinguals. More recently, L2 English sentence repetition accuracy has been explored in ‘early sequential’ bilinguals (educated only in L2 and exposed to L1 at home), in ‘late sequential’ bilinguals (educated also in L1), and in L2 monolingual children aged 9–12 years. Findings showed that only the late bilingual group shows a lower sentence repetition accuracy than the other groups, while syntactic competence was equal in early bilinguals and monolinguals [23].

Some authors have documented that sentence repetition accuracy is sensitive to language experience, suggesting the need to assess whether the use of the two languages by bilingual children is balanced or unbalanced [24]. In this sense, L2 sentence repetition skills may be assessed in L2 learners with at least 12 months of exposure to L2 [2] or in those for whom L2 is the dominant language—in terms of L2 use and exposure—in the bilingual population [23]. The variability of bilingual samples recruited and the discordance among results about L2 receptive vocabulary and expressive grammar skills require further investigation.

### 1.2. The Role of Individual and Language Exposure Factors on Receptive Vocabulary and Expressive Syntactic Abilities in L2

Now, the question is as follows: Which are the factors predicting L2 receptive vocabulary and expressive syntax in bilingual children from low-SES families and L1 minority homes? In the literature, studies distinguish child-level individual and language exposure-related factors. For receptive vocabulary, the individual factors included age at testing, gender, non-verbal cognitive functioning, and also phonological processing, whereas language exposure factors encompassed the quality and the quantity of exposure. In a longitudinal study of bilingual children with L1 Spanish and L2 American English, aged 4 to 6 years, the use of L2 tends to increase with age, whereas their L1 tends to decrease. In addition, a gender difference is found, with mothers using L2 more frequently with males than with females. However, neither the use of L2 at home nor gender differences predict L2 receptive vocabulary [25]. Thus, the use of L1 at home does not negatively influence L2 learning. Another longitudinal study [16] including bilingual children with L1 Spanish and L2 American English documents that L1 receptive vocabulary at school entry is the main factor predicting L2 receptive vocabulary at school age.

Exploring L2 receptive vocabulary in a very heterogeneous sample of 5–7-year-old children for L1 with an exposure to L2 Canadian English of almost 2 years, ref. [26] found that multiple factors, such as age at testing, phonological processing, and non-verbal cognitive functioning, explain 33% of the variance of L2 receptive vocabulary. Yet, language exposure factors, such as months of exposure and richness of exposure for L2 (the sum of frequencies with which the child is engaged in reading, stories, listening to music, TV) explain about 10% of additional variance of the L2 receptive vocabulary.

Considering another sample of bilingual 5–8-year-old children, who were exposed to L1 Vietnamese and dominant for L2 English, ref. [27] found that only the phonological processing factor explained 12% of the variance for L2 vocabulary comprehension, whereas other factors did not contribute to the prediction. Otherwise, in a sample of bilingual children aged 4 to 7 years and exposed to L1 Chinese and L2 Canadian English, ref. [28] identified more predictors of L2 receptive vocabulary. In detail, among the individual factors found were verbal processing, non-verbal cognitive functioning, and maternal education, whereas among exposure-related factors, the length of L2 exposure at school and the richness of L2 exposure at home were prevalent. 

Regarding which factors predict L2 expressive syntactic ability, some studies highlighted the role of factors related to language exposure—such as the age of onset of exposure, the percentage of L2 spoken at home, language dominance, and the length of exposure to L2—and related to L2 mastery, such as receptive vocabulary for L2. For example, the accuracy of expressive syntax in L2 English explored in a heterogeneous group for L1 children aged 5 to 8 years is predicted by L2 age of onset of exposure and the percentage of L2 spoken at home [22]. Language dominance represents the first predictor explaining syntactic accuracy for L2 German in typically developing bilingual children exposed to L1 Arabic, European Portuguese, or Turkish [29]. Therefore, the study confirms the weight of linguistic dominance on syntactic accuracy. Significant relationships between expressive syntactic performance and cumulative exposure have been found [2,30] found that language experience is the only factor impacting syntactic accuracy among early sequential bilinguals, whereas among late sequential bilinguals, there are also L2 receptive vocabulary, non-verbal cognitive functioning, and SES of the family.

Therefore, in our opinion, it is important to further analyze which factors affect L2 receptive vocabulary and expressive syntax in early sequential/simultaneous bilinguals as preschoolers and school-aged children educated only in L2 Italian and exposed to L1 from birth and L2 before the age of 4, who are very numerous in Italian schools. Moreover, considering the great impact of family SES on L2 development, it is crucial to select homogeneous samples for family SES.

### 1.3. The Aims of This Study

To our knowledge, the literature has depicted an incomplete picture of the L2 language skills among typically developing children belonging to migrant families with minority L1 and very low SES. Therefore, the aim of the current study was two-fold. First, it aimed to describe and estimate receptive vocabulary and expressive syntactic skills in L2 Italian among early sequential/simultaneous bilingual children of migrant single-mother families. In particular, we focused on families with very low SES and exposure to a minority language at home, two aspects depicted as risk factors for language development by the literature [3]. In agreement with previous evidence, we hypothesized lower performances on receptive vocabulary [15,17,18] as well as on expressive syntax in L2 (among early sequential bilinguals compared to monolinguals.

Furthermore, considering the predictive role of phonological processing in language development, we were interested to explore this competence among our participants. As a whole, evidence from the literature about phonological processing in L2 is controversial, with some studies documenting worse performances among bilinguals compared to monolinguals and other studies confirming the same abilities in both groups [22,29,31]. Moreover, ref. [32] emphasized the protective role of SES. In light of these controversial findings, we were not able to define a clear hypothesis on phonological processing.

Secondly, this study aimed to identify which individual (e.g., chronological age, sex, phonological processing, and non-verbal cognitive functioning) and language exposure (e.g., the length of L2 exposure in the educational context) factors contribute to learning L2 receptive vocabulary and expressive syntax. To our knowledge, evidence on a group of children as in this study—early sequential/simultaneous children of migrant single-parent families with low SES—is not exhaustive and therefore we would like to make a contribution to this line of research. As a whole, few studies have investigated the predictive role of individual factors (e.g., level of non-verbal cognitive and phonological processing) and factors related to language exposure for both L2 receptive vocabulary [26,27,28] and expressive syntax [22,29], but the results are not exhaustive.

## 2. Method

### 2.1. Participants

The inclusion criteria used to recruit the sample were the following: (1) children were early sequential/simultaneous bilingual in L2 Italian (they were exposed to Italian before age 4); (2) children belonged to migrant single-mother families with very low SES and supported by social services; (3) children ranged from 4 to 12 years old (the ages for which the BVL is validated [33]; (4) mothers not having received a prior diagnosis of mental disorder; and (5) children’s normal non-verbal cognitive functioning, as estimated by the Raven’s Coloured Progressive Matrices text (CPM [34,35]).

Thirty-seven cooperatives or associations located in Northern Italy were contacted by phone or email, and only five of them responded, showing interest in participating in this study. Through these organizations, we identified 60 migrant children. Thirty-one children were excluded because (1) their age was outside the age range of 4–12 years (*n* = 10); (2) they received a prior diagnosis of neurodevelopmental disorder (*n* = 8); or (3) their mothers did not attend appointments (*n* = 13). Additionally, 5 children were excluded because they were exposed to L2 after age four. Thus, the final sample consisted of 24 typical developing children (11 females; mean age = 8.13; SD = 1.89) of whom 83.3% (*n* = 20) were born in Italy. At the time of this study, three children attended pre-school, nineteen primary school, and two secondary school. Table 1 presents the sample background.

All the mothers were born outside Italy. Specifically, one mother came from South America (Brazil), eight from Africa (Ivory Coast, Gambia, Ghana, Nigeria, Morocco, Tunisia), two from Asia (i.e., Sri Lanka and Syria), and four from Eastern Europe (Albania and Moldova). They had lived in Italy for an average of 14.25 years (SD = 6.89 years; range = 4.0–32.0 years), and their average chronological age was 36.71 years (SD = 6.34 years; range = 22–48 years). They spoke 11 first languages (Ivorian French, Nigerian English, Ghanaian English, Tunisian Arabic, Moroccan Arabic, Albanian, Moldavian, Slovak, Syrian, Tamil, Mandinga, Singhalese, and Portuguese).

The maternal educational level was around 9.41 years on average (SD = 4.16 years; range = 0–16 years; 2 data points missing). Employment was present in 37.5% (*n* = 9) of them and was of ‘low profile’ (*n* = 7, cleaning company; *n* = 1, multinational technology company; *n* = 1, practicum). As emerged from a self-report ranging from 0 to 10, the Italian oral fluency of the mothers scored 8.25 in comprehension (SD = 1.89; range = 4–10) and 8.29 in production (SD = 2.07; range = 4–10). As regards the housing condition, each dyad was supported by social services and lived in mother–child communities (*n* = 12; 50%), in shared flats with one or two dyads (*n* = 2; 8.3%), or in single flats (*n* = 10; 41.7%). 

With regard to language exposure, the L1 and L2 exposure in a typical week inside and outside the home (current L1 and L2 exposure) and the length of L2 exposure to school (cumulative L2 exposure) were investigated (for details, see Section 2). Details on current L1 and cumulative L2 exposure are reported in Table 1.

### 2.2. Procedure

The research was presented to the heads of the selected organizations. After obtaining their agreement to participate in this study, the research goals were explained to the mothers who were eligible to participate in this study. If interested, mothers were asked to fill in the informed consent form. The second author of this paper carried out the interviews with the mothers and the assessment of the children’s cognitive and language skills in the spaces provided by the involved organizations. Specifically, each mother carried out the Linguistic Biography Interview [36], lasting approximately 30 min; each child completed the Raven’s Coloured Progressive Matrices test (and three subtests of the Battery for Language Assessment [35], proposed in randomized order and lasting approximately 45 min per child. The mothers’ interview and the children’s assessment were audio-recorded and transcribed verbatim. This study received full ethical approval from the Research Ethics Committee of Roma Tre University (blinded for review process).

### 2.3. Tasks

Linguistic Biography. A modified version of the semi-structured original interview by [36] was administered. In Section 1, demographic characteristics were required, including questions on the child’s health status (e.g., full-term birth, health problems) and school attendance (e.g., from what age he/she has attended school and which grade he/she currently attends), maternal education level (e.g., number of years of schooling), presence and type of maternal employment, maternal country of origin, and type of dyad housing (e.g., mother–child community, shared flat, single flat). Section 2, about the language environment, included questions about the mother’s use of language at home with the child, the percentage of current exposure to L1 and L2 in a typical week (12 h per day; Mon–Fri = 60 h; Sat/Sun = 24 h, 84 h total), calculating hours of exposure to L2 and L1 during school time and extra-school time (e.g., sports, music, religious community), and the mother’s Italian comprehension and production fluency (self-reported on a 10-step scale). We used a child’s attendance of Italian infant nursery and/or kindergarten to calculate the length of exposure to L2, expressed in years, by subtracting the child’s age of first exposure to Italian in an educational context from their current age.

Non-verbal intellectual functioning. Raven’s Coloured Progressive Matrices (CPM [34,35]) was used to assess non-verbal cognitive functioning. It includes 36 perceptual and conceptual matching items where the child must complete a matrix by choosing one of six pieces. This instrument has already been used in studies with bilingual migrant children (among others [5,37]. The child’s raw score is converted into a Z-score using Italian normative data.

As described above, for the assessment of receptive vocabulary, expressive syntax, and phonological processing, specific tests from the Language Assessment Battery for 4–12-year-olds (BVL) were used. This battery provides normative data for Italian children (*n* = 1086; age range = 4–11 years) and has been validated in Italy. Specifically, the internal consistency of the Lexical Comprehension Test of the BVL is 0.421 and 0.721 for preschoolers and school-aged children; the internal consistency of the Sentence Repetition Test of the BVL is 769 and 788 for preschoolers and school-aged children 769; the internal consistency of the Non-Word Repetition Test of the BVL is 721. Results from test–retest are good (Lexical Comprehension Test of the BVL for preschoolers, *r* = 0.639; *p* = 0.024; Lexical Comprehension Test of the BVL for school-aged children, *r* = 0.642, *p* < 0.001; Sentence Repetition Test of the BVL for preschoolers, *r* = 0.839, *p* = 0.054; *p* < 0.001; Sentence Repetition Test of the BVL for school-aged children, *r* = 0.867, *p* < 0.001; Non-Word Repetition Test of the BVL, *r* = 0.676, *p* = 0.020).

Receptive vocabulary. The Lexical Comprehension Test of the BVL (LC-BVL) was used to investigate receptive vocabulary as in previous studies [38,39]. The child is required to identify, among 4 figures, those that represent the meaning of a list of 42 target words. The words are balanced by frequency of use (very high, high, and low) for Italian. After listening to the target word, the child is presented with the target figure (corresponding to the meaning of the target word, e.g., ‘pasta/pasta’) together with the figure of a semantic distractor (e.g., of a meaning corresponding to the word semantically related to the target—e.g., panino/”sandwich”, a phonological distractor (of a meaning corresponding to the word phonologically related to the target—e.g., ‘posta/email’), and a non-relative distractor (e.g., casa/home). The figures are black-and-white drawings. The target words comprise nouns (31), verbs (10), and an adjective (1). The child has a maximum of 10 s to answer. The task is interrupted after 5 consecutive wrong answers. For each correct answer, the child receives 1 point (maximum score = 42).

Expressive syntax. Expressive syntactic abilities were assessed by the Sentence Repetition Test of the BVL (SR-BVL). The child is required to repeat 20 sentences of increasing length and complexity: 17 simple sentences (passive, e.g., il signore è tirato dalla capra/the man is pulled by the goat; negative with verb, e.g., il ragazzo non dorme/the boy does not sleep; imperative, e.g., non correre/do not run; with copular, e.g., il loro cesto è pieno di frutta/their basket is full of fruit; affirmative construction, e.g., le mamme sono contente/the mums are happy) and 3 complex sentences (split sentence, e.g., e’ Mario a finire per primo/it is Mario who finished first, and relative sentences, e.g., il gatto che corre sotto il tavolo è molto grande/the cat that runs under the table is very big). One point is scored for each correctly produced sentence (maximum score = 20). The task is interrupted after five consecutive incorrect answers. In agreement with the objective of this study, phonological/articulatory errors were not analyzed.

Phonological processing. The Non-Word Repetition Test of the BVL (NWR-BVL) was used to assess phonological processing. It includes 15 non-words composed of typical syllables of the Italian phonological system. The non-words are ordered by complexity and increasing length: monosyllables of three phonemes (e.g., bro); bisyllables of four (e.g., dego), five (e.g., permo), and six (e.g., stalmo) phonemes; trisyllables of six (e.g., livuba) and seven (e.g., vistalo) phonemes; and quadrisyllables of eight (e.g., gilovane), ten (e.g., citialesco), and eleven (e.g., qualesentro) phonemes. The non-words were spoken aloud by the second author who is a native Italian speaker. When the child correctly repeats all the phonemes of each non-word, a point is given (maximum score = 15).

### 2.4. Data Analysis

All statistical analyses were carried out with SPSS software version 25 for Windows. First, children’s performances on the BVL were considered in order to estimate language skills in L2 Italian. Second, Spearman bivariate and partial (controlling for chronological age) correlations were used to verify the associations between study variables. Finally, two hierarchical regressions were run with receptive vocabulary and expressive syntax as the outcome variables, respectively. In Step 1, age, non-verbal cognitive function, and phonological processing were entered, whereas in Step 2, length of L2 exposure at school was added.

## 3. Results

### 3.1. Children’s Performance and Monolingual Norms

Children (*n* = 24) were grouped based on their chronological age into thirteen age groups, following the suggestions provided by the authors of the BVL [33]. When more children were inserted into an age group, an aggregate score was computed for each test of the BVL (e.g., 6;0–6;5 age group). Only one participant was 12 years old and was inserted into the 11;–11.11-year age group. However, her main performance on the BVL tests was not different from those obtained by children aged 11 years. 

Children’s raw scores on the BVL—receptive vocabulary, expressive syntax, and phonological processing—were converted into zeta scores, considering mean and standard deviation from the normative sample (for details, see [33]). Zeta scores range from −2 SD to +2 SD. However, as claimed by [33], older children often obtain the maximum scores in different tests of the BVL, and in these cases it is not possible to convert raw scores into zeta scores equal to +1, +1.5, or +2. Thus, zeta scores equal to 0 are preferred. 

As shown in Table 2, the children’s mean performances were very heterogenous among the three tasks of the BVL. For the receptive vocabulary, the 7;0–7;5, 8;0–8;5, 9;6–9;11, and 11;6–11;11-year age groups scored −1.5 SD below the mean. Except for the child in the 5;5–5.11-year age group, whose score was +2 SD above the mean, all the children in the remaining age groups scored −2 DS below the mean. The performance on the expressive syntax task was −2 SD below the mean for all the age groups. The results for phonological processing indicated that the 6;0–6;5- and 11.0–11.5-year age groups scored −2 SD below the mean, the 8.0–8.5-year age group scored −1.5 SD, the 9;6–9;11-year age group scored −1 DS, whereas the remaining age groups scored average.

### 3.2. Interrelations among Individual and Language Exposure Factors

Children’s chronological age correlated positively and significantly with non-verbal cognitive functioning, measured by the Raven’s Test and all the three BVL language measures (receptive vocabulary, expressive syntax, and phonological processing). Non-verbal cognitive functioning was positively and significantly correlated with expressive syntax, receptive vocabulary, and the length of L2 exposure at school. Expressive syntax was positively correlated with receptive vocabulary and the length of L2 exposure at school which, in turn, correlated with each other. Current exposure to L1 did not correlate with any study variable. For this reason, current exposure to L1 was not inserted into regression analyses. Descriptive statistics and results from Spearman correlations are summarized in Table 3. When partial correlations were run controlling for age, receptive vocabulary was positively associated with expressive syntax (*r* = 0.73; *p* < 0.001) and the length of L2 exposure at school (*r* = 0.49; *p* < 0.05). Moreover, the length of L2 exposure at school was positively related to expressive syntax (*r* = 0.54; *p* < 0.01). No other significant associations were found.

### 3.3. Predictors of Receptive Vocabulary in L2 and Expressive Syntax in L2

Two hierarchical multiple regressions were computed considering receptive vocabulary and expressive syntax as the outcome variables, respectively. Due to the lack of significant correlations, sex and current exposure to L2 and L1 were not inserted into the regression analyses. As shown in Table 4, when control factors were entered in Step 1, the regression was significant, with phonological processing and age of the test positively predicting receptive vocabulary. The addition of the length of L2 exposure in the educational context (year) in Step 2 increased the prediction. Specifically, phonological processing and language exposure positively and significantly predicted receptive vocabulary. 

When we considered expressive syntax as the outcome variable, the regression in Step 1 was significant, with age and phonological processing as positive predictors. In Step 2, phonological processing and language exposure positively and significantly predicted expressive syntax. Details of the regression analysis are displayed in Table 5.

## 4. Discussion

The aims of the current study were (1) to explore the L2 skills of early sequential/simultaneous bilingual 5–12-year-old children of single-parent migrant households with very low SES and (2) to identify which factors impacted the acquisition of receptive vocabulary and expressive syntax in L2.

The sample consisted of typically developing bilinguals and was heterogeneous in terms of the mothers’ linguistic and cultural backgrounds—11 countries of origin and L1 minorities—but homogeneous in terms of their children’s L2 exposure. Children belonged to single-parent families with very low SES (education and profile/no occupation), assisted by Italian social services, and showing normal non-verbal cognitive functioning. 

Regarding the first objective, the results about receptive vocabulary in L2 showed that bilingual children in our sample learning a second language have lower vocabulary sizes compared to monolinguals. This finding is consistent with evidence in the literature (among others). However, as reported in [17] study, if lexical comprehension is computed by measuring the two L1 and L2 vocabularies separately and controlling for SES, sequential/simultaneous bilinguals have lower performance than L2 monolinguals, while this difference disappears if conceptual vocabulary is computed. 

As regards expressive syntactic skills, all children perform lower than same-aged monolinguals on L2. Therefore, language skills are more impaired both in terms of the number of children whose performance is not in the normal range and also in relation to the level achieved (<2 DS). Our results do not confirm [23] study which was conducted on late, early sequential bilingual children and monolingual children in which early sequential bilingual children performed as well as monolinguals. The contradiction between the results of the [23] and ours is probably determined by the sample characteristics. In [23], the sample was composed of children of second-generation migrant families with higher cognitive skills—as evaluated through a word digit span task and word reading task—than monolinguals. In addition, early and monolingual groups did not differ in SES, whereas in our study, only the early sequential bilingual group with low SES was considered and compared to monolinguals drawn from a representative sample of the Italian population with medium SES.

Results on phonological processing showed that fifteen bilingual children of our sample performed in the normal range when compared to that of the normative sample composed of monolinguals of the same age. Thus, it is evident that most L2-dominant bilingual children with a duration of L2 exposure of more than 2 years exhibit age-appropriate phonological processing, although this issue is not true for all children. In the literature, phonological processing in sequential bilingual children has been investigated with different types of non-word repetition tests (e.g., language-specific, cross-linguistic-specific test [40]. Studies have examined task performance in children with very different L1 and L2 demographics and exposure characteristics, thus leading to variability and non-uniformity in the results [22,29,31,41,42,43]. Ref. [44] showed that 71% of sequential bilinguals (L2 Chinese/Asian; L2 English/Canadian; first exposure to L2 equal to 4 years, average exposure duration 18 months, average educational level) aged 5 and 7 years perform at or above normal compared to monolinguals. We hypothesize that our children’s lower ability is justified by the fact that they come from low-SES families. In fact, ref. [32] showed that phonological processing abilities are lower in low-SES bilinguals compared to medium-SES bilinguals and L2 monolinguals with medium and low SES.

The second objective of this study was to identify which predictors among the individual and language exposure factors impacted receptive vocabulary and expressive grammar skills in L2 of early sequential/simultaneous bilingual children. We found that receptive vocabulary is predicted by phonological processing and the length of L2 exposure in L2. Our results are in line with those of [26], the phonological processing has a strong impact on language comprehension as well as the length of L2 exposure. This issue is very interesting considering the difference between our and their studies. Specifically, in [26] study, children were exposed exclusively to L1 for the first 3 years, and the first exposure to L2 occurred around age 4. In addition, the duration of exposure to L2 averaged 20 months. In [28], the children were Chinese-English, exposed to L2 from 4 years of age on average, with a length of L2 exposure of about 4 years, and with medium maternal education level. Differently, in our study, children were exposed to L2 very early, and so they were L2-dominant, and their length of L2 exposure was about 6 years on average (with some children even reaching 10 years of exposure); furthermore, mothers had low SES and low educational level.

In the present study, we also found that expressive syntax in L2 is predicted by phonological processing and the length of L2 exposure. Previous studies identified language experience as a key predictor of L2 expressive syntax [22,29,30]. In particular, ref. [22] found the first exposure to L2 to be a key predictor of L2 expressive syntax in a sample of multilingual children (i.e., children of migrants exposed to different L1s at home), aged 66 to 99 months, with a very heterogeneous first age of exposure to L2 (range = 0−81 months), a highly variable percentage of exposure to L2, and whose families had an intermediate educational level. In the study by [29], the key predictor of syntax in L2 for typically developing bilinguals was language dominance (amount of use and exposure to L1/L2), but most children were simultaneous bilinguals rather than early sequential bilinguals, with an average length of L2 exposure of 5 years and aged between about 6 and 9 years. 

Our finding that phonological processing is a predictor of the sentence repetition task has already been documented in the literature by [18] in bilingual children aged 5–8 years with L1 Kannada (a very inflectionally rich southern Indian language) and L2 English, with schooling in L1 and L2, home language exposure in L1, and with highly variable exposure to English (ranging from 1 to 6 years). Therefore, our data confirm the need to deeply investigate individual differences in expressive syntax in bilinguals, taking into account the lexical knowledge, phonological processing and language production mechanisms related to linguistic experience that affect the acquisition of this competence [18,20].

## 5. Limitations and Future Perspectives

Several limitations must be taken into account in interpreting the results of the present study. Firstly, this study is based on data of bilingual children from fragile households assisted by social services (i.e., low-SES families) but with a typical non-verbal cognitive development. However, the high rates of misdiagnosis among multilingual children with typical and atypical language development do not allow us to completely ensure our sample contains only children without language disorders. Moreover, the presence of certified diagnosis of neurodevelopmental disorder in children and mental disorder in mothers and low cooperation of mothers resulted in a substantial selection of the initial sample. Again, the choice to make the sample homogeneous from the point of view of language exposure characteristics (age of onset of L2 exposure; length of exposure to L2 in educational contexts) in order to select only early sequential/simultaneous bilinguals resulted in an additional loss of recruited children and mothers. Thus, this is a very special sample: the selection made from the sample is a limitation (small numbers) but also a strength (uniformity of the participants). Looking forward, since to our knowledge studies about L2 acquisition in bilingual children belonging to single-parent migrant households assisted by social services are scarce, it is important to pay attention not only to early sequential but also to late sequential bilinguals to capture differences in learning, as reported in the literature (among others [32]).

A further limitation of this study is the comparison between the performance of bilingual children with that of monolinguals (normative sample). Looking forward, it would be interesting to control for both SES (high vs. low levels) and bilingualism (bilinguals vs. monolinguals) separately to verify if low-SES bilinguals would perform worse than the other groups (high-SES bilinguals; low-SES monolinguals) for different language skills and not just lexical comprehension [32].

Another limitation of this study is the heterogeneity of the sample for L1. Unfortunately, the use of different L1s makes it impossible to analyze the effect of possible transference from L1 to L2 and thus how the similarity or difference between the two languages (L1 and L2) in even early sequential bilingual children may impact their language development and L2 acquisition, as demonstrated by the approach called the ‘interdependence hypothesis’ [45,46]. However, this heterogeneity depicts the actual societal picture in Italy.

## 6. Conclusions

SES family and L1 minority language are broadly considered as strong factors impacting L2 development in bilingual children. For this reason, we only focused on a specific population: early sequential/simultaneous bilinguals with very low-SES families. These characteristics constitute a key strength of our study. 

The overall results of this work, although they are to be considered with much caution given the limitations, confirm that phonological processing is the pivotal factor in L2 receptive vocabulary as well as in L2 expressive syntax in bilingual children. Language exposure variables (e.g., length of L2 exposure in educational contexts) emerge as crucial in our study too, suggesting the need for further investigations on specific characteristics of language experience.

## Figures and Tables

**Table 1:** Sample background.

Child	Gender	Birth in Italy	Age of Test (y;m)	Age of Onset to L2 (y;m)	Length of Exposure to L2 in the Educational Context (y)	Current Exposure to L1 Minority (%)	Language Spoken with the Mother
1	M	No	10;2	3;2	7	35	L2
2	F	Yes	7;5	4;0	3.5	24	Both
3	M	Yes	11;1	4;1	7	32	Both
4	M	Yes	8;1	2;1	6	32	Both
5	F	No	12;4	4;4	8	33	Both
6	M	Yes	9;6	2;6	7	22	Both
7	F	No	5;10	1;10	4	32	Both
8	M	No	7;6	3;6	4	30	Both
9	M	Yes	6;1	4;1	2	19	L2
10	M	Yes	10;4	0;4	10	17	L2
11	F	Yes	11;11	0;11	11	17	L2
12	F	Yes	6;0	1;0	5	17	L2
13	F	Yes	7;3	2;3	5	13	L2
14	M	Yes	8;7	2;7	6	36	Both
15	F	Yes	7;9	0;9	6	31	Both
16	M	Yes	9;4	2;4	7	11	L2
17	F	Yes	10;8	2;8	8	17	Both
18	F	Yes	8;6	0;6	8	11	L2
19	M	Yes	6;5	0;5	6	18	Both
20	M	Yes	6;6	0;6	6	44	Both
21	M	Yes	9;6	0;6	9	21	Both
22	M	Yes	10;8	0;8	10	30	L2
23	F	Yes	8;1	1;1	7	15	L2
24	F	Yes	8;8	2;8	6	11	Both

**Table 2:** Children’s raw scores and corresponding Z-scores per the BVL tests.

Age Group (y;m)	*n*	Receptive Vocabulary (LC-BVL)		Expressive Syntax (SR-BVL)		Phonological Processing (NWR-BVL)	
		Raw Score	Z-Score	Raw Score	Z-Score	Raw Score	Z-Score
5;5–5;11	1	24.00	+2 SD	5.00	−2 SD	13.00	0
6;0–6;5	4	18.00	−2 SD	5.00	−2 SD	8.75	−2 SD
6;6–6;11	1	21.00	−2 SD	5.00	−2 SD	14.00	0
7;0–7;5	2	27.50	−1.5 SD	9.00	−2 SD	15.00	0
7;6–7;11	2	24.50	−2 SD	6.00	−2 SD	14.00	0
8;0–8;5	2	29.00	−1.5	7.00	−2 SD	13.00	−1.5 SD
8;6–8;11	3	27.00	−2 SD	9.00	−2 SD	14.67	0
9;0–9;5	1	35.00	0	14.00	−2 SD	15.00	0
9;6–9;11	2	32.50	−1.5 SD	12.00	−2 SD	14.00	−1 SD
10;0–10;5	1	32.00	−2 SD	14.00	−2 SD	15.00	0
10;6–10;11	2	37.00	0	16.00	−2 SD	15.00	0
11;0–11;5	1	33.00	−2 SD	7.00	−2 SD	13.00	−2 SD
11;6–11;11	2	36.50	−1.5 SD	14.50	−2 SD	15.00	0

Note: (y;m) = year;month. NWR = Non-Word Repetition Test; LC = Lexical Comprehension Test; SR = Sentence Repetition Test.

**Table 3:** Bivariate correlations among study variables and descriptive statistics.

Study Variables	1.	2.	3.	4.	5.	6.	7.	8.
1. Chronological age (year)	−							
2. Sex (F = 0; M = 1)	0.03	−						
3. Non-verbal cognitive functioning (Raven’s Test)	0.60 **	−0.08	−					
4. Phonological processing (NWR-BVL)	0.44 *	−0.28	0.40	−				
5. Receptive vocabulary (LC-BVL)	0.75 ***	0.19	0.46 *	0.40	−			
6. Expressive syntax (SR-BVL)	0.74 ***	0.11	0.55 **	0.56 **	0.88 ***	−		
7. Length of L2 exposure in the educational context (year)	0.70 ***	0.07	0.49*	0.38	0.76 ***	0.77 ***	−	
8. Current exposure to L1 (%)	0.09	0.38	0.11	−0.22	−0.02	−0.10	−0.16	−
*Descriptive Statistics*								
Mean	8.13	−	0.07	13.37	9.21	28	6.6	23.65
SD	1.89	−	0.92	3.41	4.84	8.57	2.15	9.43
Range	5.1–12.4	−	−1.9–1.51	0–15	0–17	1–39	2–11	11–44

Note. NWR-BVL = Non-Word Repetition Test of the BVL (index of phonological processing); LC-BVL = Lexical Comprehension Test of the BVL (index of receptive vocabulary); SR-BVL = Sentence Repetition Test of the BVL (index of expressive syntax). * *p* < 0.05; ** *p* < 0.01; *** *p* < 0.001.

**Table 4:** Results of hierarchical regression analysis for receptive vocabulary.

Predictors	R	R^2^	ΔR^2^	B	SE	Beta	t
*Step 1*	0.86 ***	0.74					
Chronological age (year)				2.17	0.63	0.48	3.441 **
Non-verbal cognitive functioning (Raven’s Test)				−0.33	1.23	−0.03	−0.2656
Phonological processing (NWR-BVL)				1.43	0.31	0.57	4.605 ***
*Step 2*	0.90 *	0.80	0.06				
Chronological age (year)				1.328	0.65	0.29	2.041
Non-verbal cognitive functioning (Raven’s Test)				−0.25	1.09	−0.03	−0.225
Phonological processing (NWR-BVL)				1.251	0.28	0.50	4.388 ***
Length of L2 exposure in the educational context (year)				1.351	0.53	0.34	2.545 *

* *p* < 0.05; ** *p* < 0.01; *** *p* < 0.001.

**Table 5:** Results of hierarchical regression analysis for expressive syntax.

Predictors	R	R^2^	ΔR^2^	B	SE	Beta	t
*Step 1*	0.79 ***	0.62					
Chronological age (year)				1.16	0.43	0.45	2.727 *
Non-verbal cognitive functioning (Raven’s Test)				0.49	0.83	0.09	−0.593
Phonological processing (NWR-BVL)				0.62	0.21	0.44	2.965 **
*Step 2*	0.85 *	0.72	0.01				
Chronological age (year)				0.58	0.43	0.22	1.324
Non-verbal cognitive functioning (Raven’s Test)				0.55	0.73	0.10	0.753
Phonological processing (NWR-BVL)				0.50	0.19	0.35	2.604 *
Length of L2 exposure in the educational context (year)				0.94	0.35	0.42	2.648 *

* *p* < 0.05; ** *p* < 0.01; *** *p* < 0.001.

## Data Availability

The dataset used and analyzed in the current study is available from the corresponding author upon reasonable request.
